# MORTALITY RISK BY STATE-LEVEL POVERTY IN COLOMBIA AT DIFFERENT AGE GROUPS

**DOI:** 10.1093/geroni/igac059.2655

**Published:** 2022-12-20

**Authors:** Margarita Osuna, Eileen Crimmins, Jennifer Ailshire

**Affiliations:** University of Southern California Leonard Davis School of Gerontology, Santa Clarita, California, United States; University of Southern California, Los Angeles, California, United States; University of Southern California, Los Angeles, California, United States

## Abstract

Residence in high poverty states has been associated with increased mortality risk in the United States, but less attention has been paid to the relationship between state-level poverty and mortality in younger to older adults in Latin America. Poorer states in Colombia, one of the most populous and rapidly aging countries in Latin America, tend to report less access to healthcare, education, and economic opportunities. We examine the relationship between mortality and state-level poverty in Colombia by age and gender. We use data from the 2018 Colombian Census and Vital Statistics to calculate mortality levels and male-female ratios in mortality separately for three broad age categories: young (ages 20-39), middle-aged (40-69), and older adults (70 or older). We find an association between high poverty and high mortality risk among younger men, no association for middle-aged men, and a negative association between mortality risk and poverty among older men. We did not find any evidence for an association between state-level poverty and mortality at any ages. Our results highlight that, for men at older ages, poverty had a counterintuitive association with mortality. These results may be due to selective survival older ages in Colombia or to older adults who are aging into a social safety net that includes healthcare and income benefits. Future research should investigate the impact that unequal access to economic resources and distribution of health care resources has on women and men across age groups in Colombia.

